# Supplementary material for: The embryonic role of juvenile hormone in the firebrat, Thermobia domestica, reveals its function before its involvement in metamorphosis
Source: eLife. 2024 Apr 3;12:RP92643. doi: 10.7554/eLife.92643 (PMC10994664; doi:10.7554/eLife.92643)
Supplement: Figure 1—source data 1. — Age of each sample calculated from the midpoint of its 12 hr egg collection. [file elife-92643-fig1-data1.docx]

| **Embryonic age**  **hrs (days)** | **N** | **germ band** | **extended limbs** | **post katatrepsis** | **eye pigmentation** | **dorsal closure** | **shed EC1** | **resorb EEF** | **hatch** |
| --- | --- | --- | --- | --- | --- | --- | --- | --- | --- |
| 50 h (2D) | 23 | 65% | 0 |  |  |  |  |  |  |
| 62 h (2.5D) | 23 | 100% | 0 |  |  |  |  |  |  |
| 74 h (3D) | 24 | 100% | 79% | 0 |  |  |  |  |  |
| 84h (3.5D) | 25 | 100% | 100% | 0 |  |  |  |  |  |
| 96 h (4D) | 33 | 100% | 100% | 76% | 0 |  |  |  |  |
| 108 h (4.5D) | 28 | 100% | 100% | 100% | 0 |  |  |  |  |
| 121 h (5 D) | 24 | 100% | 100% | 100% | 8% |  |  |  |  |
| 133 h (5.5 D) | 23 | 100% | 100% | 100% | 48% |  |  |  |  |
| 145 h (6 D) | 23 | 100% | 100% | 100% | 95% | 0 |  |  |  |
| 157 h (6.5 D) | 38 | 100% | 100% | 100% | 100% | 0 |  |  |  |
| 169 h (7 D) | 30 | 100% | 100% | 100% | 100% | 10% | 0 |  |  |
| 180 h (7.5D) | 48 | 100% | 100% | 100% | 100% | 77% | ND | 0 |  |
| 192 h (8 D) | 37 | 100% | 100% | 100% | 98% | 98% | 71% | 3% |  |
| 204 h (8.5 D) | 26 | 100% | 100% | 100% | 100% | 100% | 85% | 15% |  |
| 216 h (9 D) | 41 | 100% | 100% | 100% | 100% | 100% | 95% | 78% | 0 |
| 229 h (9.5 D) | 28 | 100% | 100% | 100% | 100% | 100% | 100% | 96% | 0 |
| 241 h (10 D) | 50 | 100% | 100% | 100% | 100% | 100% | 100% | 100% | 0 |
| 253 h (10.5 D) | 50 | 100% | 100% | 100% | 100% | 100% | 100% | 100% | 10 |
| 265 h (11 D) | 50 | 100% | 100% | 100% | 100% | 100% | 100% | 100% | 94% |
| 277 h (11.5 D) | 50 | 100% | 100% | 100% | 100% | 100% | 100% | 100% | 100% |
|  |  |  |  |  |  |  |  |  |  |
