# Supplementary material for: The embryonic role of juvenile hormone in the firebrat, Thermobia domestica, reveals its function before its involvement in metamorphosis
Source: eLife. 2024 Apr 3;12:RP92643. doi: 10.7554/eLife.92643 (PMC10994664; doi:10.7554/eLife.92643)
Supplement: Figure 2—source data 1. — Age of samples from midpoint of 12 hr egg collections. E: embryonic age; J: age of juvenile; n.d.: below range of detectability. [file elife-92643-fig2-data1.docx]

| Sample | Age at extraction | **individuals extracted** | **pg/ml** | **fmol** | **Fmole JH III/**  **individual** |
| --- | --- | --- | --- | --- | --- |
| 001 | E_5d 9h | 196 | n.d. | 0 | 0 |
| 002 | E_5d 9h | 203 | n.d. | 0 | 0 |
| 003 | E_5d 9h | 205 | n.d. | 0 | 0 |
| 004 | E_5d 9h | 208 | n.d. | 0 | 0 |
| 005 | E_6d 10h | 202 | 97 | 54 | 0.5 |
| 006 | E_6d 10h | 212 | 5474 | 3082 | 14.5 |
| 007 | E_6d 10h | 191 | 265 | 149 | 0.8 |
| 008 | E_7d 10h | 205 | 265 | 149 | 0.7 |
| 009 | E_7d 10h | 134 | 119 | 67 | 0.5 |
| 010 | E_7d 9hr | 120 | 73 | 41 | 0.3 |
| 011 | E_8d 9h | 202 | 957 | 539 | 2.7 |
| 012 | E_8d 9h | 198 | 912 | 514 | 2.6 |
| 013 | E_8d 9h | 171 | 802 | 451 | 2.6 |
| 014 | E_9d 9hr | 198 | 1748 | 984 | 5 |
| 015 | E_9d 9hr | 192 | 1102 | 621 | 3.2 |
| 016 | E_9d 9hr | 199 | 1946 | 1096 | 5.5 |
| 017 | E_10d 9h | 204 | 4403 | 2480 | 12.2 |
| 018 | E_10d 9h | 201 | 15864 | 8933 | 44.4 |
| 019 | E_10d 9h | 201 | 21158 | 11914 | 59.3 |
| 020 | E_11d 9h | 191 | 10874 | 6123 | 32.1 |
| 021 | E_11d 9h | 209 | 7402 | 4168 | 19.9 |
| 022 | E_11d 9h | 167 | 16019 | 9021 | 54 |
| 030 | J_d0 9hr | 160 | 303 | 171 | 1.1 |
| 031 | J_d0 9hr | 150 | 125 | 70 | 0.5 |
| 032 | J_d0 9hr | 150 | 221 | 124 | 0.8 |
| 033 | J_d1 9hr | 150 | 58 | 33 | 0.2 |
| 034 | J_d1 8hr | 150 | 94 | 53 | 0.4 |
| 035 | J_d1 8hr | 150 | 51 | 29 | 0.2 |
| 036 | J_d2 8hr | 140 | 107 | 60 | 0.4 |
| 037 | J_d2 8hr | 131 | 104 | 59 | 0.5 |
| 038 | J_d2 9hr | 150 | 279 | 157 | 1 |
| 039 | J_d3 9hr | 150 | 46 | 26 | 0.2 |
| 040 | J_d3 9hr | 150 | 88 | 50 | 0.3 |
| 041 | J_d3 9hr | 140 | 53 | 30 | 0.2 |
| 042 | J_d4 9h | 75 | 85 | 48 | 0.6 |
| 043 | J_d4 9h | 75 | 103 | 58 | 0.8 |
| 044 | J_d4 9h | 75 | 121 | 68 | 0.9 |
| 045 | J_d5 8h | 75 | 109 | 61 | 0.8 |
| 046 | J_d5 8h | 75 | 169 | 95 | 1.3 |
| 047 | J_d5 8h | 75 | 89 | 50 | 0.7 |
| 048 | J_d6 9hr | 75 | 396 | 223 | 3 |
| 049 | J_d6 9hr | 75 | 383 | 215 | 2.9 |
| 050 | J_d6 8 hr | 75 | 76 | 43 | 0.6 |
| 051 | J_d7 9 hr | 75 | 139 | 78 | 1 |
| 052 | J_d7 9hr | 66 | 108 | 61 | 0.9 |
| 053 | J_d7 9hr | 66 | 73 | 41 | 0.6 |
| 054 | J_d8 9hr | 75 | 134 | 76 | 1 |
| 055 | J_d8 9hr | 75 | 156 | 88 | 1.2 |
| 056 | J_d8 9hr | 75 | n.d. | 0 | 0 |
| 057 | J_d9 - J4 | 45 | n.d. | 0 | 0 |
| 058 | J_d9 - J4 | 45 | 43 | 24 | 0.5 |
| 059 | J_d9 - J4 | 40 | n.d. | 0 | 0 |
