# Supplementary material for: The embryonic role of juvenile hormone in the firebrat, Thermobia domestica, reveals its function before its involvement in metamorphosis
Source: eLife. 2024 Apr 3;12:RP92643. doi: 10.7554/eLife.92643 (PMC10994664; doi:10.7554/eLife.92643)
Supplement: Figure 2—source data 2. — Age of samples from midpoint of 12 hr egg collections. Hatching occurred at 11.5 days after egg laying (AEL). [file elife-92643-fig2-data2.docx]

| **age (days AEL)** | **Sample 1 pg/individual** | **Sample 2 pg/individual** | **Sample 3 pg/individual** | **Sample 4 pg/individual** | **Sample 5 pg/individual** | **Average**  **pg/individual** |
| --- | --- | --- | --- | --- | --- | --- |
| 1 | 4.2 | 7.1 |  |  |  | 5.7 |
| 1.5 | 6.8 | 7.7 |  |  |  | 7.2 |
| 2 | 8 | 7.1 |  |  |  | 7.5 |
| 2.5 | 23 | 8.8 | 11.9 |  |  | 14.6 |
| 3 | 11 | 11.7 | 14.8 |  |  | 12.5 |
| 3.5 | 9.5 | 13.4 | 19.2 |  |  | 14.1 |
| 4 | 10 | 11.5 | 17.1 |  |  | 12.8 |
| 4.5 | 5.9 | 8.2 | 9.6 |  |  | 7.9 |
| 5 | 8.5 | 6.8 | 10.6 |  |  | 8.6 |
| 5.5 | 7.4 | 12.2 | 14 |  |  | 11.2 |
| 6 | 15.8 | 9.6 | 18.9 |  |  | 14.7 |
| 6.5 | 39.5 | 74.7 | 128.1 |  |  | 80.7 |
| 7 | 82.6 | 233 |  |  |  | 157.8 |
| 7.5 | 34.3 | 35 | 20.8 |  |  | 30.1 |
| 8 | 14.84 | 23 |  |  |  | 18.9 |
| 8.5 | 13.2 | 11 | 12.4 | 19.8 | 29 | 17 |
| 9 | 12.2 | 20 | 11 |  |  | 14.4 |
| 9.5 | 12.2 | 20 | 14.2 | 12.6 | 41.8 | 20.2 |
| 10 | 12.9 | 14.8 | 14.3 |  |  | 14 |
| 10.5 | 14.3 | 17.4 | 17.1 | 25 | 16.2 | 18 |
| 11 | 138.7 | 198.6 | 237.7 |  |  | 191.7 |
| 11.5 | 66.3 | 42.7 | 82.7 | 72.4 |  | 66.1 |
| 12.5 | 32.1 | 29 |  |  |  | 30.6 |
| 13.5 | 44 | 51.9 |  |  |  | 48 |
| 14.5 | 67.7 | 91.3 | 27.6 |  |  | 62.2 |
| 15.5 | 18.5 | 34.8 | 16.8 |  |  | 23.4 |
| 16.5 | 25 | 19.8 |  |  |  | 22.4 |
| 17.5 | 21.3 | 27 |  |  |  | 29 |
