# Supplementary material for: The embryonic role of juvenile hormone in the firebrat, Thermobia domestica, reveals its function before its involvement in metamorphosis
Source: eLife. 2024 Apr 3;12:RP92643. doi: 10.7554/eLife.92643 (PMC10994664; doi:10.7554/eLife.92643)
Supplement: Figure 4—source data 1. — Developmental rescue was attempted by subsequent treatment with a juvenile hormone mimic (pyriproxyfen) at the indicated developmental time thereafter. air: air appears between embryo and eggshell because of resorption of extraembryonic fluid; eye: appearance of eye pigment. Percent values are based on surviving embryos. *: these embryos blocked before dorsal closure because of the early timing of treatment with pyriproxyfen. [file elife-92643-fig4-data1.docx]

| **treatment** | **#** | **d 5.5** | **d 6.5** | **d 7.5** | **d 8.5** | **d 9.5** | **d 10.5** | **d 11.5** | **d 12.5** | **d 13.5** |
| --- | --- | --- | --- | --- | --- | --- | --- | --- | --- | --- |
| none | 30 | eye: 0 | eye: 23 [77%] | eye: 29 [97%] | air: 0 [0%]; dead: 1 | air: 8 [28%] | air: 27 [93%] | air: 29 [100%] | hatch: 21 [72%] |  |
| cyclohexane (d3.5) | 30 | eye: 0 | eye: 26 [87%] | eye: 30 [100%] | air: 0 [0%] | air: 23 [77%]; dead: 2 | air: 28 [100%] | air: 28 [100%]; hatch: 4 [14%] | hatch: 28 [100%] |  |
| 1 ug 7EP (d3.5) | 30 | eye: 0 | eye: 30 [100%] | eye: 30 [100%] | air: 0 [0%]; dead: 1 | air: 2 [7%] | air: 3 [10%]; dead: 1 | air: 4 [14%]; hatch: 1 [3%] | air: 4 [14%]; hatch: 3 [11%] |  |
| 1 ug 7EP [d3.5]; 1ng Pyri [d6.5] | 15 | eye: 0 | eye: 13 [87%] | eye: 30 [100%] | air: 0 [0%]* | air: 0 [0%]* | air: 0 [0%]* | air: 0 [0%]* | air: 0 [0%]* |  |
| 1 ug 7EP [d3.5]; 1ng Pyri [d7.5] | 30 | eye: 0 | eye: 27 [90%] | eye: 29 [97%] | air: 1 [3%]; dead: 1 | air: 11 [38%] | air: 17 [57%] | air: 17 [57%]; hatch: 10 [34%] | air: 17 [57%]; hatch 12 [41%] |  |
| 1 ug 7EP [d3.5]; 1ng Pyri [d8.5] | 30 | eye: 0 | eye: 22 [73%]; dead: 1 | eye: 27 [93%] | air: 0 [0%] | air: 16 [55%] | air: 29 [100%] | air: 29 [100%]; hatch: 1 [3%] | air: 29 [100%]; hatch: 27 [93%] |  |
| 1 ug 7EP [d3.5]; 1ng Pyri [d9.5] | 30 | eye: 0 | eye: 27 [90%] | eye: 27 [90%]; dead: 1 | air: 0 [0%] | air: 0 [0%] | air: 14 [50%] | air: 26 [93%]; hatch: 0 [0%] | air: 26 [93%]; hatch: 5 [18%] | hatch: 26 [93%] |
