# Supplementary material for: The embryonic role of juvenile hormone in the firebrat, Thermobia domestica, reveals its function before its involvement in metamorphosis
Source: eLife. 2024 Apr 3;12:RP92643. doi: 10.7554/eLife.92643 (PMC10994664; doi:10.7554/eLife.92643)
Supplement: Figure 6—source data 1. — EEF: extraembryonic fluid; pre-dorsal closure was evident by dorsal closure to the neck region, but the posterior head capsule had not fully formed. [file elife-92643-fig6-data1.docx]

| stage attained | N | Germ band | extended limb buds | post katatrepsis | eye pigment | pre-dorsal closure | dorsal closure | resorb EEF | hatch |
| --- | --- | --- | --- | --- | --- | --- | --- | --- | --- |
| age at treatment with JHm |  |  |  |  |  |  |  |  |  |
| 0.5d | 16 | 12 [75%] | 9 [56%] |  |  |  |  |  |  |
| 1.5d | 18 | 18 [100%] | 18 [100%] |  |  |  |  |  |  |
| 2.5d | 20 | 20 [100%] | 20 [100%] |  |  |  |  |  |  |
| 3.5d | 21 | 21 [100%] | 21 [100%] | 21 [100%] | 11 [52%] |  |  |  |  |
| 4.5d | 21 | 21 [100%] | 21 [100%] | 21 [100%] | 21 [100%] | 21 [100%] | 0 [0%] |  |  |
| 5.5d | 15 | 15 [100%] | 15 [100%] | 15 [100%] | 15 [100%] | 14 [94%] | 1 [6%] |  |  |
| 6.5d | 19 | 19 [100%] | 19 [100%] | 19 [100%] | 19 [100%] | 19 [100%] | 19 [100%] | 15 [79%] | 13 [68%] |
| 7.5d | 15 | 15 [100%] | 15 [100%] | 15 [100%] | 15 [100%] | 15 [100%] | 15 [100%] | 15 [100%] | 15 [100%] |
