# Supplementary material for: The embryonic role of juvenile hormone in the firebrat, Thermobia domestica, reveals its function before its involvement in metamorphosis
Source: eLife. 2024 Apr 3;12:RP92643. doi: 10.7554/eLife.92643 (PMC10994664; doi:10.7554/eLife.92643)
Supplement: Figure 7—source data 1. — N is the number of embryos scored for each treatment. [file elife-92643-fig7-data1.docx]

| **Treatment** | **score embryos** | **N** | **Average number of pHH3 positive cells/limb** | **average** |
| --- | --- | --- | --- | --- |
| **Controls: treat at 1.5 d AEL** |  |  |  |  |
| cyclohexane 1.5 d | 2.5 d AEL | 3 | 11.3, 12.7, 9 | 11 |
| cyclohexane 1.5 d | 3.5 d AEL | 13 | 12.8, 17.6, 13.5, 17.7, 16. 10, 15, 18, 12.5, 22, 12, 16, 16.5. | 15.3 |
| cyclohexane 1.5 d | 4.5 d AEL | 11 | 21.3, 29.2, 22.5, 24, 25, 29.3, 23, 24.3, 23, 26, 33.5 | 25.6 |
| cyclohexane 1.5 d | 5.5 d AEL | 10 | 20, 22.3, 21, 20.3, 22.7, 27.3, 25.7, 31, 29, 19.5 | 23.9 |
| cyclohexane 1.5 d | 6.5 d AEL | 6 | 11.3, 11, 14, 10.6, 15.3, 14.3 | 12.8 |
| cyclohexane 1.5 d | 7.5 d AEL | 4 | 7.3, 8, 7.3, 7, 3 | 7.5 |
| **JHm: treat at 1.5 d AEL** |  |  |  |  |
| JHm 1.5 d | 2.5 d AEL | 3 | 14, 13, 10.3 | 12.4 |
| JHm 1.5 d | 3.5 d AEL | 11 | 17, 15.5, 16.4, 14.7, 16.7, 15.5, 4.8, 13.3, 14, 11, 5.8 | 13.2 |
| JHm 1.5 d | 4.5 d AEL | 11 | 2.7, 3.7, 3.2, 1., 0., 1.7, 0.3, 0, 1.5, 1.7, 0.7 | 1.5 |
| JHm 1.5 d | 5.5 d AEL | 13 | 7, 9, 4.5, 6.3, 2, 5.3, 2, 0.3, 0, 0.3, 0.2, 3.3, 0 | 3.1 |
| JHm 1.5 d | 6.5 d AEL | 14 | 5.3, 0, 4.3, 0.6, 1.5, 0.6, 1.5, 0, 0, 0, 0.3, 0, 4, 1.6, 0 | 1.4 |
| JHm 1.5 d | 7.5 d AEL | 8 | 0,0.7, 0, 0.7, 0, 0.3, 0, 0, | 0.2 |
